# CD36 Induces Inflammation by Promoting Ferroptosis in Pancreas, Epididymal Adipose Tissue, and Adipose Tissue Macrophages in Obesity-Related Severe Acute Pancreatitis

**DOI:** 10.3390/ijms26083482

**Published:** 2025-04-08

**Authors:** Ruoyi Zhang, Xin Ling, Xianwen Guo, Zhen Ding

**Affiliations:** Department of Gastroenterology, The First Affiliated Hospital, Sun Yat-Sen University, Guangzhou 510080, China; zhangruoyi_dsa@163.com (R.Z.); guoxw26@mail.sysu.edu.cn (X.G.)

**Keywords:** obesity, severe acute pancreatitis, epididymal adipose tissue, adipose tissue macrophages, ferroptosis

## Abstract

Severe acute pancreatitis (SAP) is mainly triggered by the abnormal activation of pancreatic enzymes. Obesity acts as an independent risk factor for SAP; however, the underlying mechanism has not been fully elucidated. In this study, SAP models were established in mice with normal and high-fat diets. Subsequently, this study examined ferroptosis and inflammatory markers in pancreas and epididymal adipose tissues. To mimic obesity-related SAP in adipose tissue macrophages (ATMs), lipopolysaccharide (LPS) and palmitic acid (PA) were introduced, and alterations in ferroptosis and inflammation were assessed. To elucidate the regulatory role of cluster of differentiation 36 (CD36) in ferroptosis, liproxstatin-1 (Lip-1) and sulfosuccinimidyl oleate sodium (SSO) were utilized for in-depth analysis in the pancreas, epididymal adipose tissues, and ATMs. Our findings suggest that obesity aggravates ferroptosis in pancreas tissue, epididymal adipose tissues, and ATMs during SAP, as evidenced by increased lipid peroxidation, elevated Fe^2+^ levels, and alterations in ferroptosis markers, while these alterations were regained by Lip-1. Notably, CD36 levels were significantly increased in pancreatic tissue, epididymal adipose tissue, and ATMs, indicating that CD36 promotes ferroptosis and induces inflammation. SSO treatment alleviated changes in ferroptosis markers and reduced inflammation. Western blot results showed that CD36 promoted ferroptosis through the acyl-CoA synthetase long-chain family member 4 (ACSL4)/glutathione peroxidase 4 (GPX4) axis in pancreatic tissue, while a similar regulatory role was mediated by the ferritin heavy chain 1 (FTH1)/GPX4 axis and ATMs. These findings demonstrate that CD36 induces inflammation by facilitating ferroptosis in pancreas tissue, epididymal adipose tissue, and ATMs in obesity-related SAP. The inhibition of CD36 could provide novel viewpoints for the prevention and treatment of obesity-related SAP.

## 1. Introduction

Acute pancreatitis (AP), a prevalent gastrointestinal disorder, primarily arises from the abnormal activation of pancreatic enzymes that initiate autodigestion within the pancreas and adjacent organs [1]. In recent years, growing evidence has demonstrated that obesity is not only an independent risk factor for the onset of AP but also significantly influences its disease progression and clinical outcomes. Clinical data reveal that among AP patients, the proportion of obese individuals developing severe acute pancreatitis (SAP) is markedly higher than that of non-obese individuals, accounting for approximately 30–40% of SAP cases. Patients with obesity-associated SAP exhibit a significantly increased incidence of complications, including multiple organ dysfunction syndrome (MODS), pancreatic necrosis, and infectious complications [2,3]. The underlying mechanism lies in the excessive release of free fatty acids (FFAs) from visceral adipose tissue in obese patients. These FFAs accumulate in the pancreas and exacerbate the severity of AP by inducing oxidative stress, activating inflammatory signaling pathways, and promoting damage to pancreatic acinar cells [4,5,6,7].

Ferroptosis, a tightly regulated type of programmed cell death, is characterized by the abnormal accumulation of iron-dependent lipid peroxidation products [8]. Ferroptosis is linked to various immune diseases, including nonalcoholic steatohepatitis [9], diabetes [10], neuroinflammation [11], and rheumatoid arthritis [12]. Ferroptosis may trigger harmful inflammatory responses, ultimately leading to cellular membrane disruption and the release of damage-associated molecular patterns (DAMPs) [13]. Emerging evidence suggests that trypsin exacerbated pancreatitis by promoting ferroptosis in acinar cells [14]. In mouse models of hyperlipidemic acute pancreatitis, inhibiting ferroptosis has been shown to effectively attenuate the inflammatory response [15]. A critical limitation in the field is the lack of preclinical models that faithfully replicate obesity-related SAP. Additionally, the role of ferroptosis in pancreatic and epididymal adipose tissue inflammation remains poorly defined.

Obesity is a metabolic disorder characterized by chronic low-grade inflammation, with key features including abnormal lipid accumulation in epididymal adipose tissue and significant macrophage infiltration, which releases cytokines and inflammatory mediators into the bloodstream [16,17]. These macrophages possess the capability to differentiate into pro-inflammatory M1 and anti-inflammatory M2 phenotypes in response to changes in the local microenvironment, thereby influencing inflammatory responses through the regulation of inflammatory cytokine release [18]. Notably, in obese states, the number of adipose tissue macrophages (ATMs) increases dramatically from the normal range of 5–10% to over 50% [19]. and diet-induced obesity promotes a shift from the protective M2 phenotype to the inflammatory M1 phenotype [20]. This shift likely impacts ferroptosis and inflammation in epididymal adipose tissue. Given the significant infiltration of M1-polarized macrophages in obese adipose tissue and their well-characterized pro-inflammatory properties, we hypothesize that ATMs may play a pivotal role in mediating the systemic inflammatory response in obesity-related SAP. However, the specific mechanistic role of ATMs in obesity-related SAP has remained poorly characterized, with limited understanding of how ATMs orchestrate disease progression through modulating ferroptosis and inflammatory cascades.

Cluster of differentiation 36 (CD36), a crucial receptor for long-chain fatty acid uptake and lipid metabolism [21,22], has emerged as a therapeutic target for a range of lipid metabolism-related disorders, such as non-alcoholic fatty liver disease [23], atherosclerosis [24], and diabetic nephropathy [25]. Recent studies indicate that CD36 activation exacerbates metabolic dysfunction-associated steatotic liver disease in mouse models of type 2 diabetes [26]. Furthermore, a contemporary study has indicated that osteoprotegerin can modulate CD36 transcription via the ERK-PPARγ signaling pathway, thereby fostering hepatic steatosis [27]. Furthermore, CD36 is highly expressed in various immune cells and plays a critical role in modulating inflammatory responses by regulating key inflammatory signaling pathways. Given the role of role in inflammation and lipid metabolism, it is plausible that it also regulates ferroptosis and inflammation in obesity-associated acute pancreatitis. Nevertheless, the precise molecular mechanisms governing CD36-mediated regulation of ferroptosis and inflammatory responses across pancreas, epididymal adipose, and ATM compartments in obesity-related SAP have yet to be fully elucidated.

This study aims to investigate the role of ferroptosis in the progression of obesity-related SAP, focusing on its impact on the inflammatory responses of pancreatic tissue, epididymal adipose tissue, and ATMs. By systematically screening and validating CD36 as a key upstream regulator of ferroptosis, our findings provide potential therapeutic targets and theoretical foundations for alleviating inflammatory damage in pancreatic tissue, epididymal adipose tissue, and ATMs. These results offer novel scientific insights for the prevention and treatment strategies of obesity-related SAP.

## 2. Results

### 2.1. Obesity Aggravated Systemic Inflammation and Organ Injury During SAP

After 16 weeks, in contrast to the Control group, mice in the HFD group presented a remarkable increase in body weight and raised serum TG and FFAs, verifying the successful construction of the obese mouse model (Figure S1).

H&E staining revealed that in the Control group, the acinar cells were highly organized and closely packed, presenting a distinct lobular structure. In contrast, the HFD group exhibited slightly dilated lobular septa with a loose structure.

**Figure 1:**
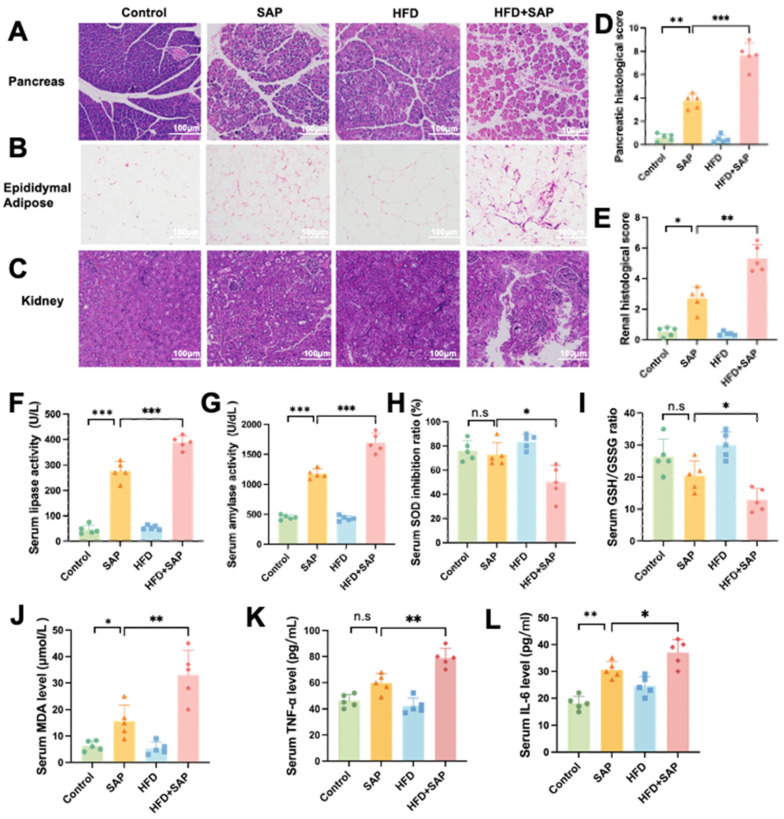
Obesity aggravated systemic inflammation and organ injury during SAP. (**A**) H&E staining of the pancreas, (**B**) epididymal adipose tissue, and (**C**) kidney. (**D**) Pancreas pathological score. (**E**) Renal pathological score. (**F**) Serum lipase activity. (**G**) Serum amylase activity. (**H**) Serum SOD inhibition ratio. (**I**) Serum GSH/GSSG ratio. (**J**) Serum MDA level. (**K**) The levels of TNFα and (**L**) IL-6 in serum. Data are shown as means ± SD (*n* = 5). * *p* < 0.05, ** *p* < 0.01, *** *p* < 0.001, n.s: no significant difference.

The SAP group exhibited significant broadening of the pancreatic stroma, swollen acinar cells, dilated lobular septa, and infiltration of inflammatory cells. Notably, in the HFD+SAP group, there was overt widening of the pancreatic stroma, distinct dilation of both lobular and acinar septa, extensive infiltration of inflammatory cells, sheet-like acinar cell necrosis, and interlobular septa hemorrhage (Figure 1A). Moreover, in comparison with the SAP group, the HFD+SAP group manifested sheet-like necrotic adipocytes, enhanced infiltration of inflammatory cells, and an increase in red blood cells (Figure 1B). Histopathological sections of renal tissue revealed that, in contrast to the SAP group, the HFD+SAP group presented more severe adhesion of glomerular capsular lumens, augmented hemorrhage and inflammatory cell infiltration, and extensive necrosis of renal tubular cells (Figure 1C). Compared with the SAP group, the pathological scores of the pancreas and kidney in the HFD+SAP group were significantly higher. Compared with the Control group, the SAP group exhibited significantly increased histopathological scores in pancreatic and renal tissues, confirming the successful establishment of the SAP model. (Figure 1D,E). In contrast to the SAP group, serum lipase (Figure 1F) and amylase activities (Figure 1G) in the HFD+SAP group were significantly elevated. Compared with the SAP group, the SOD inhibition ratio (Figure 1H) and GSH/GSSH ratio (Figure 1I) in the HFD+SAP group were significantly reduced, while the MDA level (Figure 1J) was significantly increased. Additionally, serum inflammatory factors were measured. The results showed that, compared with the SAP group, TNFα and IL-6 in the HFD+SAP group were significantly increased (Figure 1K). The SAP group exhibited significantly elevated serum levels of amylase and lipase along with increased MDA and IL-6 concentrations compared to the Control group. These results suggest that obesity aggravated oxidative stress, systemic inflammation, and organ injury during SAP.

### 2.2. Obesity Aggravated Ferroptosis in Pancreatic Tissue During SAP

**Figure 2:**
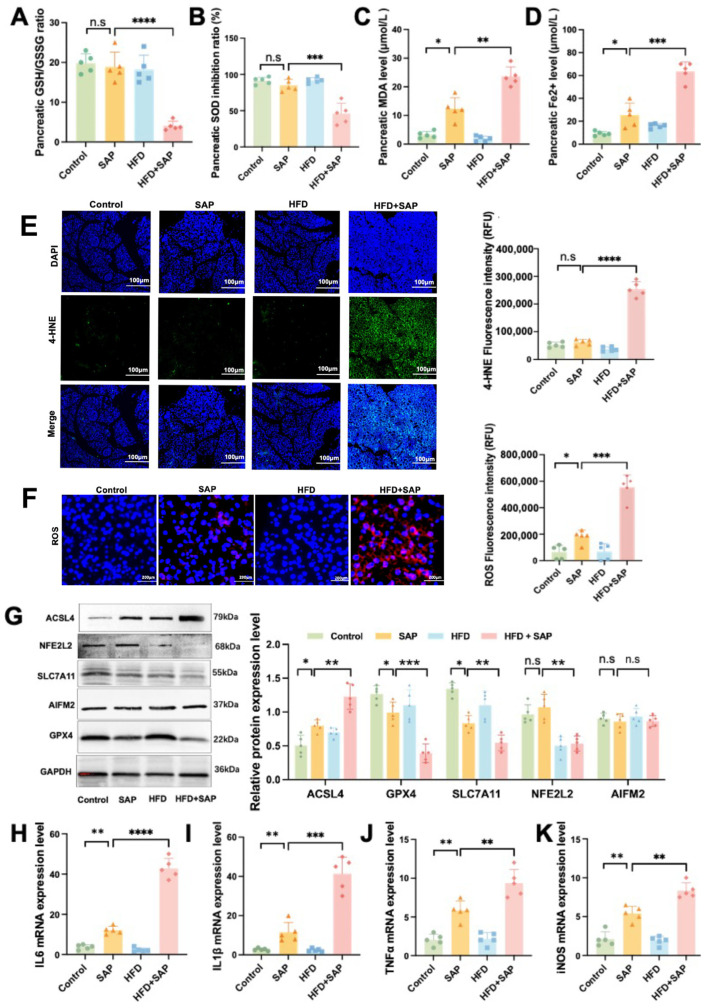
Obesity aggravated ferroptosis in pancreatic tissue during SAP. (**A**) GSH/GSSG ratio, (**B**) SOD inhibition ratio, (**C**) MDA level, and (**D**) Fe^2+^ level in the pancreatic tissue. (**E**) Immunofluorescence staining of 4-HNE and (**F**) ROS in the pancreatic tissue. (**G**) The protein levels of ACSL4, GPX4, SLC7A11, NFE2L2, and AIFM2 in pancreatic tissue. The mRNA expression levels of (**H**) IL-6, (**I**) IL-1β, (**J**) TNFα, and (**K**) iNOS in pancreatic tissue. Data are shown as means ± SD (*n* = 5). * *p* < 0.05, ** *p* < 0.01, *** *p* < 0.001, **** *p* < 0.0001, n.s: no significant difference.

The results showed that, compared with the SAP group, the GSH/GSSG ratio (Figure 2A) and SOD inhibition ratio (Figure 2B) were reduced in the HFD+SAP group, while MDA (Figure 2C) and Fe^2^⁺ levels (Figure 2D) were elevated in pancreatic tissue of the HFD+SAP group. Immunofluorescence results showed that the levels of 4-HNE (Figure 2E) and cytoplasmic ROS in pancreatic tissue in the HFD+SAP group were significantly higher than those in the SAP group (Figure 2F). Furthermore, we evaluated the levels of ferroptosis-associated marker proteins in pancreatic tissue. Western blot analysis demonstrated that, compared with the SAP group, the protein level of acyl-CoA synthetase long-chain family member 4 (ACSL4) was significantly elevated in the HFD+SAP group, whereas the protein levels of nuclear factor erythroid 2-related factor 2 (NFE2L2), solute carrier family 7 member 11 (SLC7A11), and glutathione peroxidase 4 (GPX4) were markedly reduced. (Figure 2K). RT-qPCR results showed obviously higher mRNA expression of IL-6 (Figure 2H), interleukin-1 beta (IL-1β; Figure 2I), tumor necrosis factor alpha (TNF-α; Figure 2J), and inducible nitric oxide synthase (iNOS; Figure 2K) in the HFD+SAP group compared with the SAP group. Compared to the control group, SAP model mice exhibited significantly increased levels of MDA, Fe^2^⁺, and ROS in pancreatic tissue, along with markedly upregulated protein expression of ACSL4 and downregulated levels of both GPX4 and SLC7A11. Concurrently, mRNA expression of multiple proinflammatory cytokines was substantially elevated in the SAP group. These results suggest that obesity aggravated oxidative stress and ferroptosis in pancreatic tissue during SAP and activated the GPX4-dependent classical ferroptosis signaling pathway.

### 2.3. Obesity Aggravated Ferroptosis in Epididymal Adipose Tissue and ATMs During SAP

**Figure 3:**
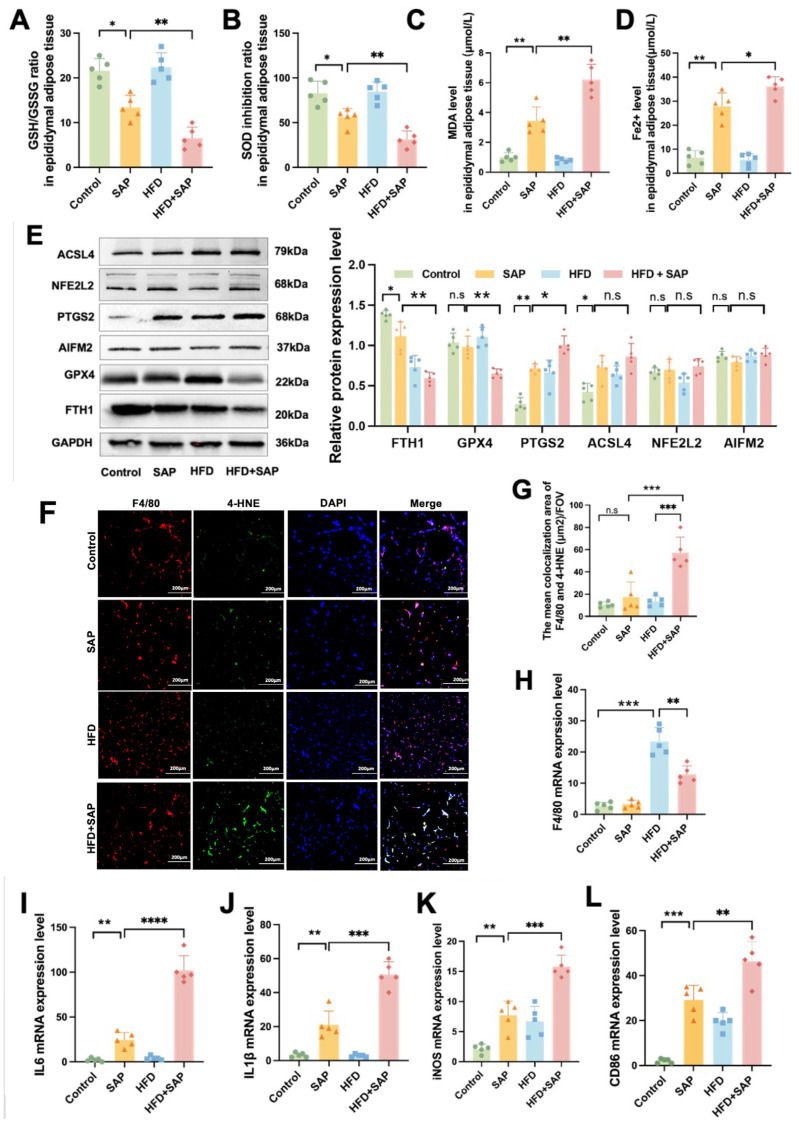
Obesity Aggravated Ferroptosis in Epididymal Adipose Tissue and ATMs during SAP. (**A**) The ratio of GSH to GSSG, (**B**) the inhibition ratio of SOD, (**C**) the level of MDA, and (**D**) the level of Fe^2+^ in epididymal adipose tissue. (**E**) The protein levels of ACSL4, NFE2L2, PTGS2, AIFM2, GPX4, and FTH1 in epididymal adipose tissue. (**F**) Immunofluorescence co-localization of 4-HNE and F4/80 in epididymal adipose tissue. (**G**) The protein levels and (**H**) the mRNA expression of F4/80 in epididymal adipose tissue. The mRNA expression levels of (**I**) IL-6, (**J**) IL-1β, (**K**) iNOS, and (**L**) CD86 in epididymal adipose tissue. Data are presented as means ± SD (*n* = 5). * *p* < 0.05, ** *p* < 0.01, *** *p* < 0.001, **** *p* < 0.0001, n.s: no significant difference.

The results showed that, compared to the SAP group, the HFD+SAP group exhibited a decrease in the GSH/GSSG ratio (Figure 3A) and a lower SOD inhibition ratio (Figure 3B). Simultaneously, there was an increase in MDA level (Figure 3C) and an elevated Fe^2^⁺ level (Figure 3D) in the epididymal adipose tissue of the HFD+SAP group. Western blot results showed that, compared with the SAP group, the protein level of Prostaglandin-Endoperoxide Synthase 2 (PTGS2) was significantly increased in the epididymal adipose tissue of the HFD+SAP group, while the protein levels of Ferritin Heavy Chain 1 (FTH1) and GPX4 were decreased markedly, and the protein levels of ACSL4, NFE2L2, and Apoptosis-Inducing Factor Mitochondria-Associated 2 (AIFM2) were not significantly different (Figure 3E). F4/80 (Adgre1/EMR1) is primarily expressed on tissue-resident macrophages, a widely used surface marker for identifying murine macrophages. Immunofluorescence results showed a significant increase in the co-localization area of 4-HNE and F4/80 in adipose tissue of the HFD+SAP group (Figure 3F,G), indicating that ferroptosis predominantly occurred in macrophages in epididymal adipose tissue. Furthermore, the mRNA expression levels of F4/80 were significantly higher in the adipose tissue of the HFD+SAP group compared to the SAP group (Figure 3H). RT-qPCR analysis showed that the mRNA expression levels of IL-6 (Figure 3I), IL-1β (Figure 3J), iNOS (Figure 3K), and Cluster of Differentiation 86 (CD86) (Figure 3L) were significantly increased in the HFD+SAP group compared to the SAP group. Compared to the control group, SAP model mice exhibited a significantly reduced GSH/GSSG ratio and SOD activity, along with markedly elevated levels of MDA and Fe^2^⁺ in epididymal adipose tissue. Furthermore, these mice showed upregulated protein expression of PTGS2 and ACSL4, coupled with downregulated FTH1 levels. Concurrently, mRNA expression of multiple proinflammatory cytokines was significantly elevated. These results demonstrate that obesity exacerbates ferroptosis and inflammatory responses in epididymal adipose tissue during SAP and may promote ferroptosis in ATMs. Obesity likely aggravates tissue inflammation by enhancing ferroptosis in macrophages within epididymal adipose tissue during SAP. Additionally, obesity activates the GPX4-dependent ferroptosis signaling pathway in epididymal adipose tissue during SAP.

### 2.4. Lip-1 Alleviates Ferroptosis and Inflammation in the Pancreas and Epididymal Adipose Tissues in Obesity-Related SAP

**Figure 4:**
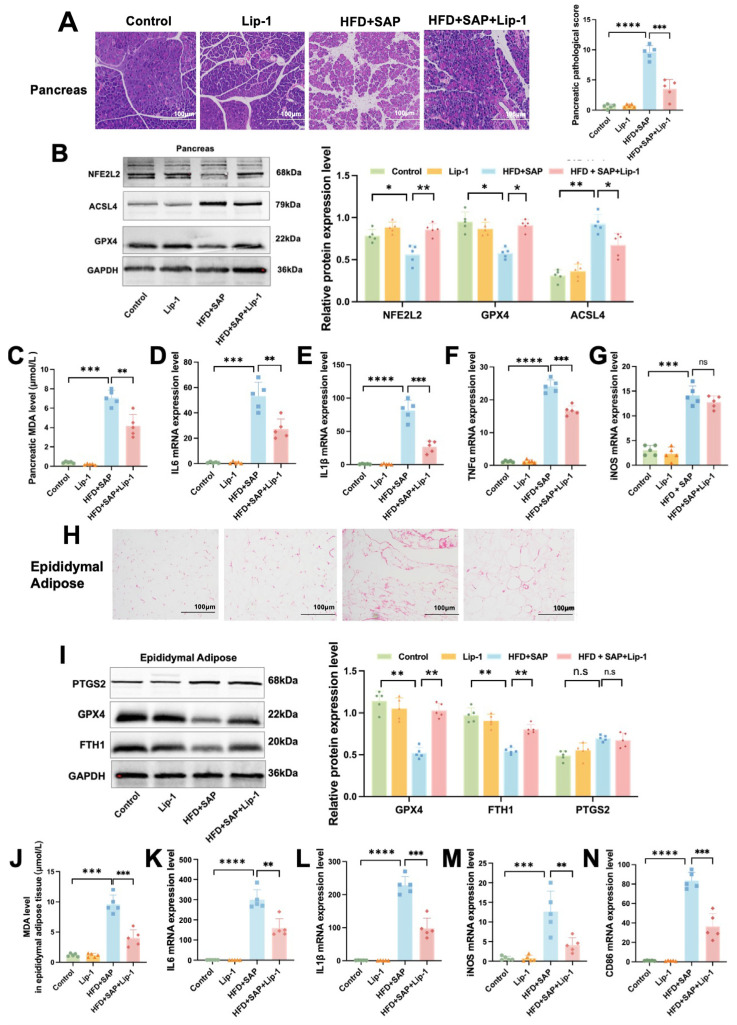
Lip-1 mitigates ferroptosis and inflammation in the pancreas and epididymal adipose tissues in obesity-related SAP. (**A**) H&E staining of pancreatic tissue was conducted. (**B**) The protein levels of ACSL4, NFE2L2, and GPX4 in pancreatic tissue were determined. (**C**) MDA levels in pancreatic tissue were measured. The mRNA expression levels of (**D**) IL-6, (**E**) IL-1β, (**F**) TNFα, and (**G**) iNOS in pancreatic tissue were evaluated. (**H**) H&E staining of epididymal adipose tissue was performed. (**I**) The protein levels of PTGS2, FTH1, and GPX4 in epididymal adipose tissue were analyzed. (**J**) MDA levels in epididymal adipose tissue were detected. The mRNA expression levels of (**K**) IL-6, (**L**) IL-1β, (**M**) TNFα, and (**N**) iNOS in epididymal adipose tissue were assessed. Data are presented as means ± SD (*n* = 5). * *p* < 0.05, ** *p* < 0.01, *** *p* < 0.001, **** *p* < 0.0001, n.s: no significant difference.

To investigate the impacts of Lip-1 on ferroptosis and inflammatory response in pancreatic tissue caused by HFD and SAP, we employed Lip-1 to assess the ferroptosis and inflammation-related indicators. H&E staining revealed that the acinar cells in both the Control and Lip-1 groups were closely arranged, and the lobular structure was clearly distinguishable. In contrast, the HFD+SAP group witnessed a considerable increase in the interval between lobules and acini, along with severe inflammatory cell infiltration and hemorrhage, and remarkable necrosis of acinar cells. Nevertheless, in the HFD+SAP+Lip-1 group, these pathological changes were significantly mitigated (Figure 4A). Western blot outcomes indicated that Lip-1 reversed the down-regulation of GPX4 and NFE2L2 protein levels and the up-regulation of ACSL4 protein level induced by HFD and SAP in pancreatic tissue (Figure 4B). Our result demonstrated that Lip-1 suppressed the increase in MDA level (Figure 4C), as well as the mRNA expression levels of IL-6 (Figure 4D), IL-1β (Figure 4E), TNFα (Figure 4F), and iNOS (Figure 4G) induced by HFD and SAP in pancreatic tissue. Compared to the control group, HFD+SAP model mice demonstrated significantly increased pancreatic histopathological scores accompanied by markedly reduced protein expression of NFE2L2 and GPX4, elevated ACSL4 levels, and substantially higher MDA content along with upregulated mRNA expression of multiple proinflammatory cytokines.

Furthermore, this study also probed into the impacts of Lip-1 on the ferroptosis and inflammation in epididymal adipose tissue brought about by HFD and SAP. As depicted in Figure 4H, the treatment of Lip-1 mitigated adipocyte necrosis and inflammatory cell infiltration and augmented bleeding in the HF+SAP group. The results of Western blot demonstrated that Lip-1 reversed the down-regulation of GPX4 and FTH1 protein levels caused by HFD and SAP in epididymal adipose tissue, yet there was no notable difference in the PTGS2 protein level (Figure 4I). In line with the discoveries in pancreatic tissue, the outcomes from the analysis of epididymal adipose tissue indicated that Lip-1 restrained the increase in MDA level (Figure 4J), as well as the mRNA expression levels of IL-6 (Figure 4K), IL-1β (Figure 4L), iNOS (Figure 4M), and CD86 (Figure 4N) induced by HFD and SAP. Compared to the control group, HFD+SAP mice exhibited significantly reduced protein expression of GPX4 and FTH1, along with markedly elevated MDA levels and upregulated mRNA expression of multiple proinflammatory cytokines in epididymal adipose tissue. The above results indicate that the ferroptosis inhibitor Lip-1 alleviates inflammatory damage in pancreatic and epididymal adipose tissues in obesity-related SAP by inhibiting the ferroptosis process.

### 2.5. CD36 Induces Inflammation by Promoting Ferroptosis in Pancreatic Tissue During Obesity-Related SAP

**Figure 5:**
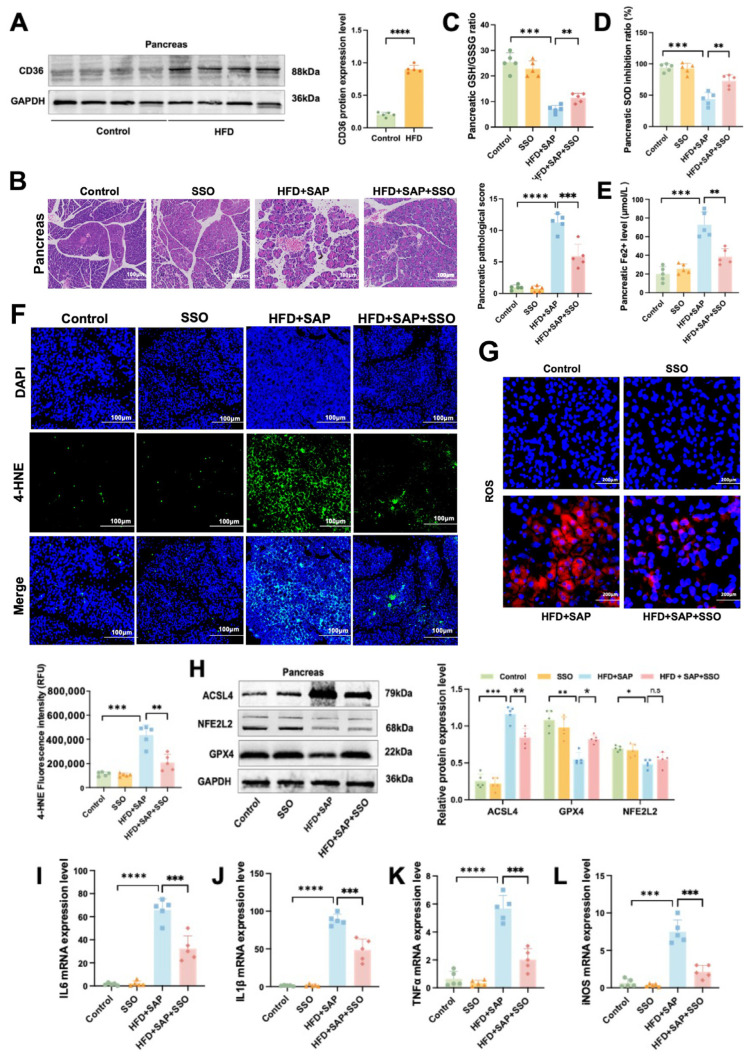
CD36 induces inflammation by promoting ferroptosis in pancreatic tissue during obesity-related SAP. (**A**) The protein levels of CD36 in pancreatic tissue. (**B**) H&E staining of pancreatic tissue. (**C**) GSH/GSSG ratio, (**D**) SOD inhibition ratio, and (**E**) Fe^2+^ level in pancreatic tissue. (**F**) Immunofluorescence staining of 4-HNE and (**G**) ROS in pancreatic tissue. (**H**) The protein levels of ACSL4, NFE2L2, SLC7A11, and GPX4 in pancreatic tissue. The mRNA expression levels of (**I**) IL-6, (**J**) IL-1β, (**K**) TNFα, and (**L**) iNOS in epididymal adipose tissue. Data are shown as means ± SD (*n* = 5). * *p* < 0.05, ** *p* < 0.01, *** *p* < 0.001, **** *p* < 0.0001, n.s: no significant difference.

Western blot results showed a significant increase in CD36 protein levels within the pancreatic tissue of the HFD group compared to the Control group (Figure 5A). As can be seen in Figure 5B, pathological injury in pancreatic tissue of obesity-related SAP mice was alleviated following SSO treatment. In addition, SSO treatment effectively suppressed the decrease in the GSH/GSSH ratio (Figure 5C) and SOD inhibition ratio (Figure 5D) and the increase in Fe^2+^ levels (Figure 5E) in pancreatic tissue induced by HFD and SAP. Immunofluorescence analysis showed that the fluorescence signal of 4-HNE (Figure 5F) and ROS (Figure 5G) was enhanced in the HFD+SAP group, but this signal enhancement was suppressed by SSO treatment. Moreover, Western blot analysis of the pancreatic tissue showed that the HFD+SAP-induced decrease in GPX4 protein level and increase in ACSL4 protein level were both reversed by SSO (Figure 5H). RT-PCR results from pancreatic tissue showed that SSO inhibited the elevation of IL-6 (Figure 5I), IL-1β (Figure 5J), TNFα (Figure 5K), and iNOS (Figure 5L) mRNA expression levels induced by HFD and SAP. Compared to the control group, HFD+SAP mice exhibited significantly aggravated pancreatic histopathological damage, accompanied by a marked reduction in the GSH/GSSG ratio and SOD activity, along with elevated Fe^2^⁺ content and increased fluorescence intensity of 4-HNE and ROS. These changes were associated with upregulated protein expression of ACSL4 and downregulated levels of both GPX4 and NFE2L2, while mRNA expression of multiple proinflammatory cytokines was substantially increased. The above results indicate that CD36 promotes ferroptosis-mediated inflammatory responses in pancreatic tissue during obesity-related SAP, and SSO significantly alleviates this process by inhibiting CD36 function. Furthermore, CD36 likely regulates the ferroptosis process in pancreatic tissue through the ACSL4/GPX4 axis.

### 2.6. CD36 Induces Inflammation by Promoting Ferroptosis in Epididymal Adipose Tissue and ATMs During Obesity-Related SAP

**Figure 6:**
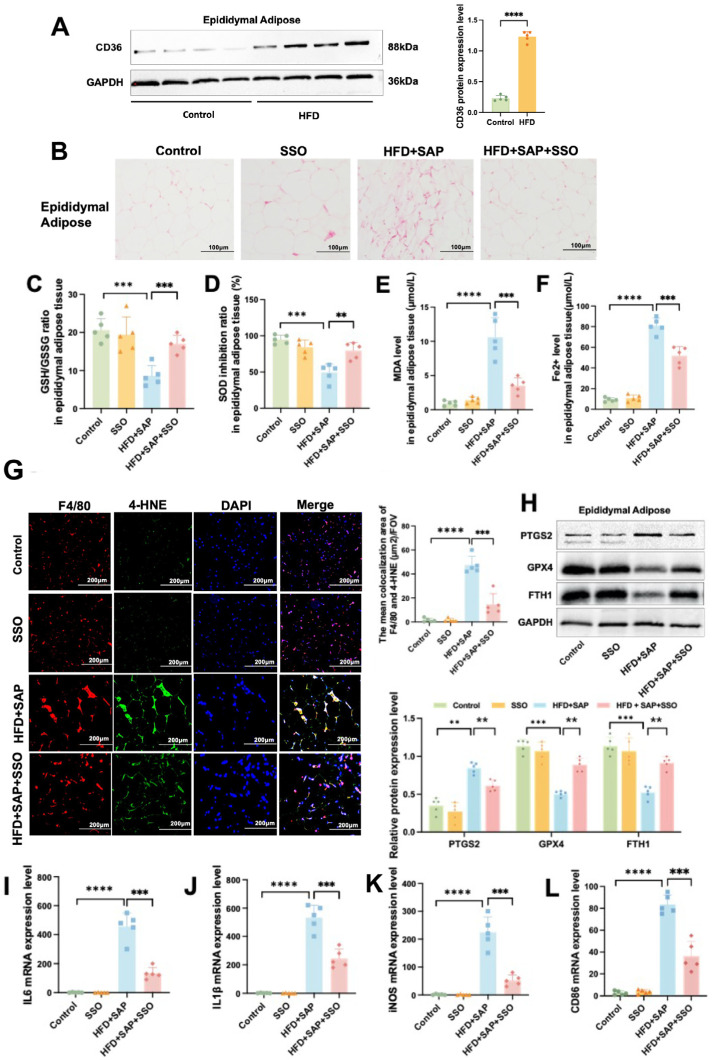
CD36 Induces Inflammation by Promoting Ferroptosis in Epididymal Adipose Tissue and ATMs during Obesity-related SAP. (**A**) The protein quantities of CD36 in epididymal adipose tissue. (**B**) H&E staining of the epididymal adipose tissue. (**C**) GSH/GSSG ratio, (**D**) SOD inhibition ratio, (**E**) MDA level, and (**F**) Fe^2+^ level in epididymal adipose tissue. (**G**) Immunofluorescence co-localization of F4/80 and 4-HNE in epididymal adipose tissue. (**H**) The protein levels of PTGS2, FTH1, and GPX4 in epididymal adipose tissue. The mRNA expression levels of (**I**) IL-6, (**J**) IL-1β, (**K**) iNOS, and (**L**) CD86 in epididymal adipose tissue. Data are presented as means ± SD (*n* = 5). ** *p* < 0.01, *** *p* < 0.001, **** *p* < 0.0001.

Western blot results showed that CD36 protein level was significantly elevated in the HFD group compared to the Control group (Figure 6A). The adipocytes in the HFD+SAP group showed necrosis, increased inflammatory cell infiltration, and bleeding, and SSO treatment alleviated these pathological changes (Figure 6B). SSO treatment mitigated the reduction in the GSH/GSSH ratio (Figure 6C) and SOD inhibition ratio (Figure 6D), while also reversing the elevation of MDA (Figure 6E) and Fe^2+^ levels (Figure 6F) that were prompted by the combination of HFD and SAP in the epididymal adipose tissue. Immunofluorescence results showed that SSO treatment alleviated the significant increase in the co-localization area of 4-HNE and F4/80 in adipose tissue of the HFD+SAP group (Figure 6G), indicating that CD36 was involved in the regulation of ferroptosis in epididymal adipose tissue. Additionally, Western blot results showed that SSO mitigated the reduction in GPX4 and FTH1 protein levels and the elevation in PTGS2 protein level induced by the combination of HFD and SAP in the epididymal adipose tissue (Figure 6H). RT-qPCR analysis of the epididymal adipose tissue showed that SSO treatment suppressed the increase in mRNA expression levels of IL-6 (Figure 6I), IL-1β (Figure 6J), iNOS (Figure 6K), and CD86 (Figure 6L) that were induced by the combined effects of HFD and SAP. These findings suggest that CD36 induces inflammation by promoting macrophage ferroptosis in epididymal adipose tissue during obesity-associated SAP. Compared to the control group, HFD+SAP mice demonstrated significantly impaired redox homeostasis in epididymal adipose tissue, as evidenced by a reduced GSH/GSSG ratio and decreased SOD activity, along with markedly elevated oxidative stress markers, including MDA, Fe^2^⁺, and 4-HNE fluorescence intensity. These changes were accompanied by dysregulated expression of ferroptosis-related proteins, with significantly downregulated GPX4 and FTH1 levels and upregulated PTGS2, while mRNA expression of multiple proinflammatory cytokines was substantially increased.

These results indicate that CD36 promotes ferroptosis-induced inflammatory responses in epididymal adipose tissue during obesity-related SAP, and SSO significantly alleviates this process by inhibiting CD36 function, as well as reducing ferroptosis in ATMs. In obesity-related SAP, CD36 likely induces tissue inflammation by promoting ferroptosis in macrophages within epididymal adipose tissue. Furthermore, CD36 may regulate the ferroptosis process in adipose tissue and ATMs through the FTH1/GPX4 axis.

### 2.7. In Vitro Validation of Ferroptosis and Inflammatory Responses in ATMs During Obesity-Related SAP

To simulate the microenvironment of ATMs in vitro under disease conditions, we used LPS to mimic the systemic inflammatory environment in obesity-related SAP and PA and to replicate the high-lipid microenvironment in epididymal adipose tissue. By stimulating mouse macrophages with LPS and PA individually or in combination, we assessed their functional changes.

**Figure 7:**
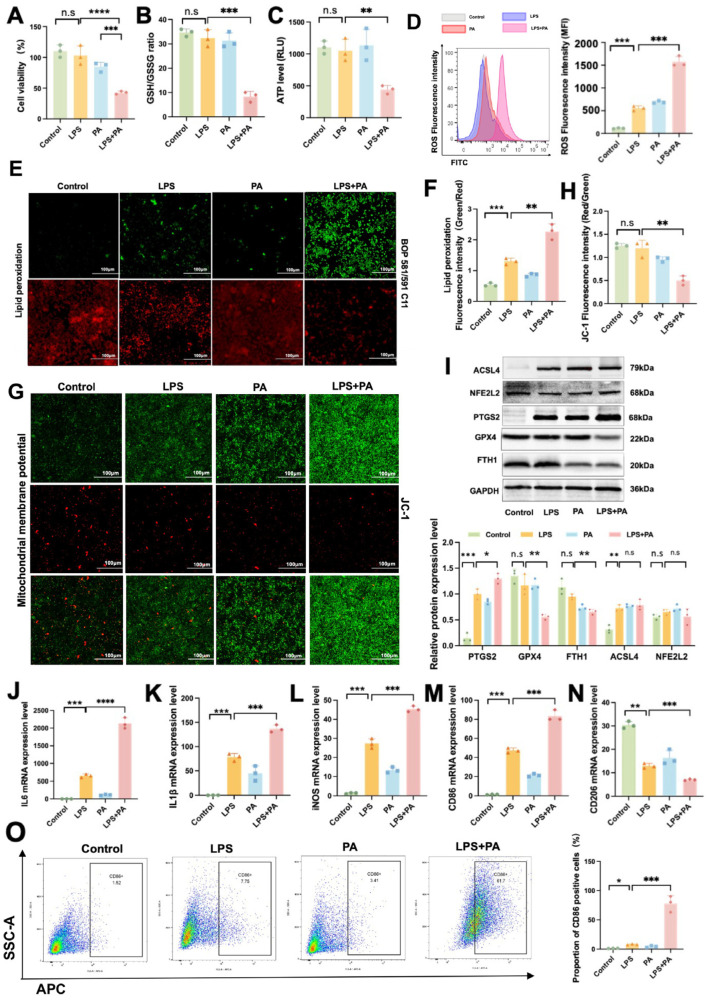
In Vitro Validation of Ferroptosis and Inflammatory Responses in ATMs During Obesity-related SAP. (**A**) Cell viability. (**B**) GSH/GSSG ratio, (**C**) ATP level, and (**D**) ROS level in ATMs. (**E**) BODIPY 581/591 C11 staining was used to detect lipid peroxidation, and (**F**) quantitative analysis. (**G**) JC-1 staining was used to detect MMP level and (**H**) quantitative analysis. (**I**) The protein expression levels of ACSL4, NFE2L2, PTGS2, FTH1, and GPX4 in ATMs. The mRNA expression levels of (**J**) IL-6, (**K**) IL-1β, (**L**) iNOS, (**M**) CD86, and (**N**) CD206 in ATMs. (**O**) The proportion of CD86-positive cells was measured using flow cytometry. Data are shown as means ± SD (*n* = 3). * *p* < 0.05, ** *p* < 0.01, *** *p* < 0.001, **** *p* < 0.0001, n.s: no significant difference.

The cell viability results revealed a marked decrease in the viability of cells in the LPS+PA group compared to the LPS group (Figure 7A). Specifically, when comparing the LPS+PA group to the LPS group, a significant reduction was observed in the GSH/GSSG ratio (Figure 7B), ATP levels (Figure 7C), and MMP levels (Figure 7G,H) within macrophages. Conversely, there was a notable increase in ROS levels (Figure 7D) and lipid peroxidation (Figure 7E,F). Co-treatment with LPS and PA aggravated the LPS-induced increase of PTGS2 protein level and the decrease of GPX4 and FTH1 protein levels in macrophages. (Figure 7I). Furthermore, the LPS+PA group exhibited peak levels of various pro-inflammatory factors, including IL-6, IL-1β, and iNOS (Figure 7J–L). Furthermore, the results showed that there was a significant increase in the proportion of M1-polarized macrophages in the LPS+PA group compared to the LPS group (Figure 7M–O). Compared to control macrophages, LPS-stimulated macrophages demonstrated significantly enhanced ROS fluorescence intensity and elevated lipid peroxidation content, coupled with upregulated protein expression of PTGS2 and ACSL4, along with markedly increased mRNA levels of multiple proinflammatory cytokines. The above results indicate that co-treatment with LPS and PA exacerbates oxidative stress, ferroptosis, and inflammatory responses in macrophages and activates the GPX4-dependent ferroptosis signaling pathway.

### 2.8. Lip-1 Alleviates Ferroptosis and Inflammation in ATMs in Obesity-Related SAP

**Figure 8:**
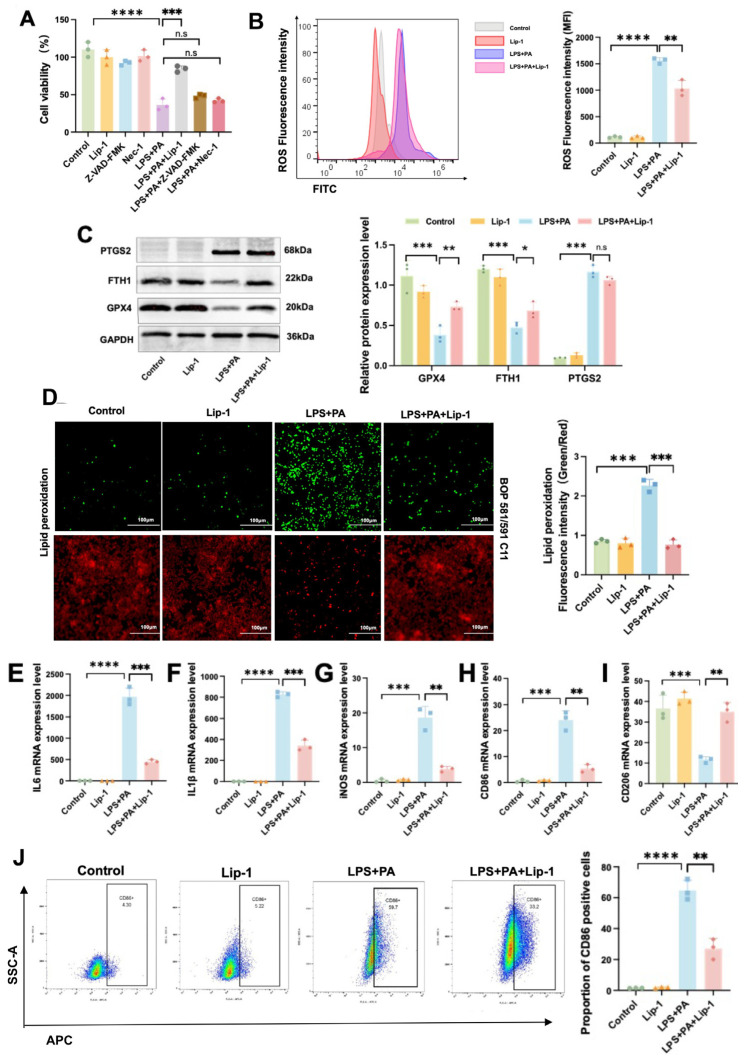
Lip-1 alleviates ferroptosis and inflammation in ATMs in obesity-related SAP. (**A**) Cell viability was assessed after treatment with Lip-1, Z-VAD-FMK, and Nec-1, respectively. (**B**) The fluorescence intensity of ROS was measured using flow cytometry. (**C**) The protein levels of PTGS2, FTH1, and GPX4 in ATMs. (**D**) Lipid peroxidation levels. The mRNA expression levels of (**E**) IL-6, (**F**) IL-1β, (**G**) iNOS, (**H**) CD86, and (**I**) CD206 in ATMs. (**J**) The proportion of CD86-positive cells was measured using flow cytometry. Data are shown as means ± SD (*n* = 3). * *p* < 0.05, ** *p* < 0.01, *** *p* < 0.001, **** *p* < 0.0001, n.s: no significant difference.

To further confirm that ferroptosis is the main mode of death in ATMs, we pretreated the cells with Lip-1, Z-VAD-FMK, and Nec-1. The CCK-8 assay showed that Lip-1 significantly alleviated the decrease in cell viability in the LPS+PA environment, while Z-VAD-FMK and Nec-1 had no significant effect (Figure 8A). Compared with the LPS+PA group, Macrophages of the LPS+PA+Lip-1 group significantly increased the level of ROS (Figure 8B). Western blot results showed that Lip-1 alleviated the decrease in GPX4 and FTH1 protein levels in LPS- and PA-induced macrophages, but there was no significant difference in PTGS2 protein level (Figure 8C). Lip-1 treatment suppressed BODIPY 581/591 C11 signal enhancement induced by LPS and PA (Figure 8D). Lip-1 alleviated the increased levels of various pro-inflammatory factors (IL-6, IL-1β, TNF, iNOS) in the LPS+PA group (Figure 8E–G). In addition, the results showed that Lip-1 mitigated the increase in the proportion of M1-polarized macrophages induced by LPS and PA (Figure 8H–J). Compared to control macrophages, LPS+PA co-treated macrophages exhibited significantly reduced cell viability accompanied by markedly increased ROS fluorescence intensity and elevated lipid peroxide content. These changes were associated with downregulated protein expression of GPX4 and FTH1, along with upregulated PTGS2 levels and substantially increased mRNA expression of multiple proinflammatory cytokines. These results suggest that ferroptosis is the primary mode of cell death in macrophages under co-treatment with LPS and PA. Additionally, inhibition of ferroptosis using Lip-1 significantly alleviates inflammatory responses and reduces the proportion of M1 polarization in macrophages.

### 2.9. CD36 Induces Inflammation by Promoting Ferroptosis in ATMs in Obesity-Related SAP

**Figure 9:**
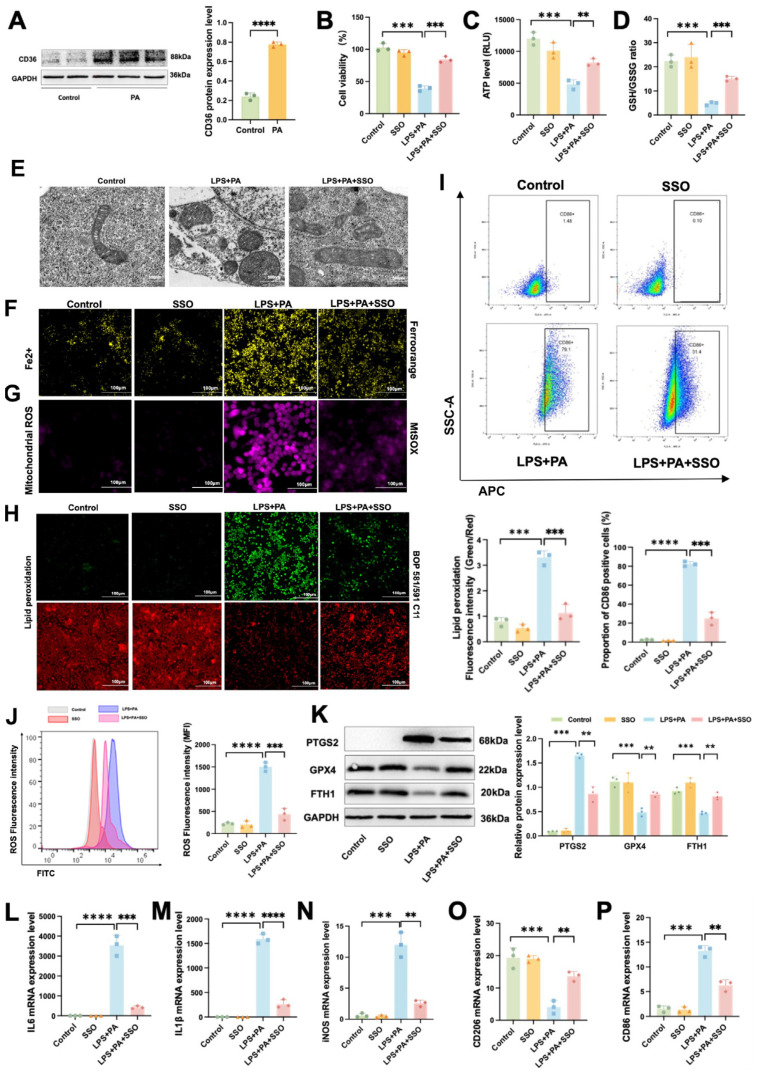
CD36 induces inflammation by promoting ferroptosis in ATMs in obesity-related SAP. (**A**) The protein levels of CD36 in ATMs. (**B**) Cell viability, (**C**) ATP level, and (**D**) GSH/GSSG ratio were assessed in ATMs after treatment with SSO. (**E**) The representative TEM images of ATMs. (**F**) The proportion of CD86-positive cells was measured using flow cytometry. Immunofluorescence staining of (**G**) Fe^2+^ and (**H**) mitochondrial ROS levels in ATMs. (**I**) Lipid peroxidation levels. (**J**) The fluorescence intensity of ROS was measured using flow cytometry. (**K**) The protein levels of PTGS2, FTH1, and GPX4 in macrophage. The mRNA expression levels of (**L**) IL-6, (**M**) IL-1β, (**N**) iNOS, (**O**) CD206, and (**P**) CD86 in macrophage. Data are shown as means ± SD (*n* = 3). ** *p* < 0.01, *** *p* < 0.001, **** *p* < 0.0001.

Western blot results showed that CD36 protein level was significantly elevated in the Control group compared to the PA group (Figure 9A). Thus, we speculate that CD36 is involved in the regulation of ATM ferroptosis. Therefore, we pretreated macrophages with SSO to explore the regulatory effect of CD36 on ferroptosis. The CCK-8 assay showed that SSO significantly alleviated the decrease in cell viability induced by LPS and PA (Figure 9B). Compared to the LPS+PA group, Macrophages in the LPS+PA+SSO group exhibited a notable decline in ATP level (Figure 9C) and GSH/GSSG ratio (Figure 9D). TEM results showed that SSO significantly reduced mitochondrial swelling and cristae breakage induced by LPS and PA in macrophages (Figure 9E). Immunofluorescence analysis showed that the fluorescence signal of Fe^2+^ (Figure 9F) and Mitochondrial ROS (Figure 9G) was enhanced in the LPS+PA group, but this signal enhancement was inhibited by SSO treatment. Conversely, these macrophages showed a marked increase in lipid peroxidation (Figure 9H) and ROS level (Figure 9J). In addition, the results showed that SSO mitigated the increase in the proportion of M1-polarized macrophages induced by LPS and PA (Figure 9I,O,P). As shown in Figure 9K, the increased PTGS2 and decreased GPX4 and FTH1 protein levels induced by LPS and PA were regained by SSO. Moreover, compared with the LPS+PA group, SSO mitigated the increase in the mRNA expression levels of IL-6 (Figure 9L), IL-1β (Figure 9M), and iNOS (Figure 9N) induced by LPS and PA in macrophages. Compared to control macrophages, the LPS+PA co-treated group exhibited significantly reduced cellular viability accompanied by decreased ATP content and GSH/GSSG ratio, along with markedly elevated lipid peroxidation levels and ROS fluorescence intensity. These metabolic alterations were associated with upregulated PTGS2 protein expression and downregulated levels of both GPX4 and FTH1, concurrent with substantially increased mRNA expression of multiple proinflammatory cytokines.

These results indicate that CD36 mediates inflammatory responses and increases the proportion of M1 polarization in macrophages by promoting ferroptosis. Furthermore, CD36 may regulate the ferroptosis process in ATMs through the FTH1/GPX4 axis.

## 3. Materials and Methods

### 3.1. Experimental Animals

Male C57BL/6 mice (4 weeks old) were procured from Zhuhai Bioscience Company in China. All the animals were kept in a standard specific-pathogen-free (SPF) laboratory with a 12 h light/dark cycle and had unrestricted access to water and food.

### 3.2. Animal Grouping and Treatment

To investigate the influence of obesity on SAP-induced ferroptosis, twenty C57BL/6 mice were randomly divided into 4 groups (*n* = 5): the Control group, the SAP group, the high-fat diet (HFD) group, and the HFD+SAP group. Mice in the Control and SAP groups were provided with a standard normal diet (12.11% kcal fat, D12079, Research Diets, Inc., New Brunswick, NJ, USA) for 16 weeks, while those in the HFD and HFD+SAP groups were given HFD for 16 weeks (60% kcal fat, D12451, Research Diets, Inc.) to build an obesity model. On the first day of week 21, mice in the SAP and HFD+SAP groups received seven intraperitoneal injections of 50 µg/kg cerulein (HY-A0190, MCE, Monmouth Junction, NJ, USA) every 1 h. Simultaneously with the last cerulein injection, 10 mg/kg of lipopolysaccharide (LPS, L2880, LABLEAD, Beijing, China) was administered intraperitoneally to induce the SAP model. Cerulein and LPS were diluted with normal saline.

To further investigate the impact of ferroptosis on obesity-related SAP, the ferroptosis inhibitor Liproxstatin-1 (Lip-1, HY-12726, MCE, Monmouth Junction, NJ, USA) was utilized. Twenty C57BL/6 mice were randomly assigned to 4 groups (*n* = 5): the Control group, the HFD+SAP group, the HFD+SAP+Lip-1 group, and the Lip-1 group. Mice in the Lip-1 group were given a normal diet for 16 weeks. Moreover, mice in the Lip-1 and HFD+SAP+Lip-1 groups were administered an intraperitoneal injection of 10 mg/kg Lip-1 1 h before the induction of the SAP model. Lip-1 was diluted with polyethylene glycol 300 (PEG300). Mice in the Control and HFD+SAP groups were injected intraperitoneally with an equal volume of PEG300.

To investigate the regulatory function of CD36 in ferroptosis and inflammation related to obesity-associated SAP, the CD36 inhibitor sulfosuccinimidyl oleate sodium (SSO, HY-112847A, MCE, Monmouth Junction, NJ, USA) was employed. Twenty C57BL/6 mice were randomly divided into 4 groups (*n* = 5): the Control group, the HFD+SAP group, the HFD+SAP+SSO group, and the SSO group. Mice in the SSO group were given a normal diet for 16 weeks. Furthermore, mice in the SSO and HFD+SAP+SSO groups were administered an intraperitoneal injection of 50 mg/kg SSO two hours before the induction of the SAP model. SSO was diluted with PEG300. Mice in the Control and HFD+SAP groups were injected intraperitoneally with an equal volume of PEG300.

All mice were put to death through cervical dislocation 24 h after the final LPS injection. Blood was gathered by inserting a capillary into the orbital sinus and centrifuged at 3000 r/min for 10 min to separate the serum. The pancreatic tissue and the epididymal adipose tissue were obtained for subsequent investigations.

### 3.3. Enzyme Activities and Proinflammatory Cytokine Detection

The reaction mixture was kept at 37 °C for 1 to 2 h. Subsequently, the absorbance of each sample was measured at the specified wavelength with a microplate reader. Eventually, the activities of the enzymes (amylase and lipase), along with concentrations of the proinflammatory cytokines tumor necrosis factor alpha (TNF-α) and interleukin-6 (IL-6), were calculated using standard calibration curves.

### 3.4. Serum Triglycerides and Free Fatty Acids Levels Detection

All procedures were strictly followed according to the manufacturer’s instructions. The serum, after collection, was added to specific assay reagents for free fatty acids (FFAs, MM-0326M1, MEIMIAN, Yancheng, Jiangsu, China) and triglycerides (TG, SEM-JM-02911M1, JINGMEI, Beijing, China), and their concentrations were calculated using standard curves based on absorbance measurements following incubation and colorimetric reactions, respectively.

### 3.5. Hematoxylin-Eosin Staining

The pancreatic tissue and epididymal adipose tissue were fixed with 4% paraformaldehyde and then embedded in paraffin. After deparaffinization and washing, the sections were subjected to hematoxylin and eosin (H&E) staining. Schmidt’s pathology scale was employed to quantitatively evaluate the severity of tissue injury, and the pathology scores were determined in a blinded manner.

### 3.6. Immunofluorescence Staining

Paraffin-embedded sections of pancreatic and epididymal adipose tissue were dewaxed by xylene and subsequently rehydrated through a series of fractional alcohols. Antigen retrieval was accomplished by using either a citrate or EDTA buffer. Subsequently, the sections were blocked with serum or bovine serum albumin (BSA) and incubated overnight at 4 °C with a primary antibody specific to 4-hydroxynonenal (4-HNE, MAB3249-SP, R&D Systems, Minneapolis, MN, USA).

Following washing, a fluorophore-conjugated secondary antibody (SA00001-1, Proteintech, Rosemont, IL, USA) was applied. The nuclei are then counterstained with DAPI (BL739B, Biosharp, Hefei, Anhui, China) for enhanced visualization.

Frozen sections of pancreatic tissue were fixed and rinsed with PBS. Subsequently, the sections were incubated with a Reactive Oxygen Species (ROS)-sensitive fluorescent probe (DHE, HY-D0079, MCE, Monmouth Junction, NJ, USA) at 37 °C in the darkness. After rinsing, the sections were mounted and inspected under a fluorescence microscope for observation.

### 3.7. Measurement of Oxidative Stress Indicators

The malondialdehyde (MDA) assay kit, the glutathione/glutathione disulfide (GSH/GSSG) quantification kit (G263, Dojindo, Kumamoto, Japan), and the superoxide dismutase (SOD) assay kit (S311, Dojindo, Kumamoto, Japan) were used to measure MDA level, GSH level, and SOD inhibition ratio in serum, pancreatic and epididymal adipose tissue, strictly following the manufacturer’s instructions.

### 3.8. Tissue Ferrous Ions (Fe^2+^) Level Assay

The pancreatic and epididymal adipose tissues were homogenized and mixed with a ferrous ion-specific chromogenic reagent (ferrozine), according to the reagent kit instructions (I291, Dojindo, Kumamoto, Japan). Following incubation, the absorbance at 562 nm was determined, and the Fe^2^⁺ concentration was computed by means of a standard curve.

### 3.9. Cell Culture and Treatment

The mouse macrophage cell line RAW264.7 (CL-0190, Pricella, Wuhan, Hubei, China) was cultured in DMEM medium with 10% fetal bovine serum. Palmitic acid (PA) was the primary FFA released by adipose tissue and was used to simulate the high-fat environment in obese adipose tissue in vitro [28,29]. LPS was used to replicate the systemic inflammatory conditions of SAP. PA was dissolved in BSA, while LPS was dissolved in normal saline. In the LPS group, RAW264.7 cells were exposed to 0.1 mg/L LPS for a duration of 24 h. Cells in the PA group were treated with 500 µM PA for 24 h. Cells in the LPS+PA group were co-treated with 0.1 mg/L LPS and 500 µM PA for 24 h. The control group of RAW264.7 cells was treated with an equal volume of BSA for 24 h.

To explore the modes of cell death induced by LPS and PA in RAW264.7 cells, we employed ferroptosis inhibitor Lip-1, apoptosis inhibitor Z-VAD-FMK, and necrosis inhibitor Necrostatin-1 (Nec-1). Cells in the LPS+PA+Z-VAD-FMK group were pretreated with 40 µM Z-VAD-FMK for 24 h, followed by co-treatment with 0.1 mg/L LPS and 500 µM PA for an additional 24 h. Cells in the LPS+PA+Necrostatin-1 group were pretreated with 30 µM Necrostatin-1 for 24 h prior to the addition of LPS and PA. Cells in the LPS+PA+Lip-1 group were pretreated with 10 µM Lip-1 for 24 h and then co-treated with 0.1 mg/L LPS and 500 µM PA for another 24 h. Lip-1, Z-VAD-FMK, and Necrostatin-1 were dissolved in Dimethyl sulfoxide (DMSO). The Control group underwent identical treatment with an equal volume of BSA and DMSO.

To investigate the regulatory function of CD36 in the ferroptosis of macrophages, we employed the CD36 inhibitor SSO. The cells in the LPS+PA+SSO group were pretreated with 40 µM SSO for 1 h and then co-treated with LPS and PA for 24 h. SSO was dissolved in DMSO. The Control group was given an equal volume of BSA and DMSO.

### 3.10. Cell Viability Assay

RAW264.7 cells were inoculated in 96-well plates for cultivation and exposed to diverse drug treatments. Subsequently, the cells were incubated with CCK-8 reagent (CCK-8, C0038, Beyotime, Shanghai, China) at 37 °C for 1–2 h. Absorbance values were then measured by a microplate reader.

### 3.11. Lipid Peroxidation Assay

The BODIPY 581/591 C11 probe (L248, Dojindo, Kumamoto, Japan) was employed for fluorescence and quantitative determination of cellular lipid peroxides. At the beginning, RAW264.7 cells were inoculated onto 6-well plates and 96-well plates and treated with various drugs. Next, the BODIPY 581/591 C11 probe was diluted 1000 times in serum-free medium and added to the cells, which were then incubated in the dark for 30 min to guarantee sufficient binding of the probe to intracellular lipid peroxides. After the incubation, the cells in the 6-well plate were observed under a fluorescence microscope. The fluorescence intensity of the cells in 96-well plates was detected by a fluorescent absorbance reader.

### 3.12. Intracellular Iron Assay

The intracellular iron levels were determined by employing the sensitive fluorescence probe FerroOrange (F374, Dojindo, Kumamoto, Japan). RAW264.7 cells were subjected to various drugs and then washed with PBS. The FerroOrange probe was diluted 1000-fold with serum-free medium and added to the cells, followed by incubation for 1 h. The cells were observed under a fluorescence microscope.

### 3.13. Mitochondrial ROS Assay

RAW264.7 cells underwent treatment with diverse drugs followed by washing with PBS. Subsequently, the MtSOX probe (R254, Dojindo, Kumamoto, Japan) was diluted 1000-fold in serum-free medium, added to the cells, and incubated in the dark for 1 h. After three additional PBS washes, the cells were examined under a fluorescence microscope.

### 3.14. Mitochondrial Membrane Potential Assay

For JC-1 staining (MT09, Dojindo, Kumamoto, Japan), the probe was diluted 500× in serum-free medium. Post-drug treatment, RAW264.7 cells were washed 3 times with PBS and incubated with the diluted probe for 30 min. After removing the probe-containing medium, the cells were visualized under a fluorescence microscope.

### 3.15. Adenosine Triphosphate (ATP) Level Detection

Following completion of drug treatment in 96-well plates, 10 μL of ATP working solution (E-BC-F002; Elabscience Biotechnology, Wuhan, Hubei, China) was aseptically added to each well containing culture medium. After incubation at 37 °C with 5% CO_2_ for 1 h, relative light units (RLUs) were quantified using a multimode microplate reader configured for chemiluminescence detection.

### 3.16. Flow Cytometry

Following treatment, the cells were gathered, centrifuged at 300× *g* for 5 min at 4 °C, and resuspended in PBS. After the ultimate centrifugation, a 1:1000 ROS probe (S0033S, Beyotime, Shanghai, China) was introduced. The cells were incubated for 30 min, centrifuged, resuspended in PBS, and analyzed by flow cytometry through the FITC channel. Dead and adherent cells were excluded, and the ROS fluorescence intensity was analyzed using histograms.

After treatment, cells were resuspended in PBS, centrifuged at 300× *g* for 5 min, and adjusted to 1 × 10^6^ cells/mL. For staining, 100 µL of cell suspension was mixed with 6 µL of APC-conjugated anti-CD86 antibody and incubated at room temperature in the dark for 30 min. Unstained cells served as the negative control. Cells were washed, centrifuged, and resuspended in 200 µL PBS for flow cytometry analysis using the APC channel. Data were analyzed with FlowJo (version 10.8.1) to determine the percentage of CD86-positive cells.

### 3.17. Transmission Electron Microscopy

RAW264.7 cells were fixed in 2.5% glutaric dialdehyde to preserve their ultrastructure. Following post-fixation with osmium tetroxide, the cells were subjected to dehydration via a graded ethanol sequence and embedded in resin. Ultrathin sections (500 nm) were acquired by using an ultramicrotome and placed on copper grids. These sections were stained with uranyl acetate and lead citrate to achieve better contrast. The samples were then examined under a transmission electron microscope (TEM) to analyze their ultrastructure.

### 3.18. Western Blot Assay (WB)

The pancreatic tissue, the epididymal adipose tissue, and RAW264.7 cells were homogenized, and the proteins were extracted by using RIPA lysis buffer (P0013, Beyotime, Shanghai, China). Following centrifugation at 12,000× *g* for 10 min at 4 °C, the protein concentration of the supernatant was determined with a bicinchoninic acid assay kit (A53225, Thermo Scientific, Waltham, MA, USA). Equal quantities of the protein samples were separated through SDS (P0014D, Beyotime, Shanghai, China) and then transferred onto polyvinylidene difluoride (PVDF) membranes (CAS#:24937-79-9, Sigma-Aldrich, Burlington, MA, USA). These membranes were then incubated overnight with the primary antibody against ACSL4 (ab155282, Abcam, Cambridge, UK), GPX4 (ab125066, Abcam, Cambridge, UK), FTH1 (ab183781, Abcam, Cambridge, UK), CD36 (ab252922, Abcam, Cambridge, UK), and SLC7A11 (ab307601, Abcam, Cambridge, UK). Subsequently, the membranes were treated with secondary antibodies (PR30011, Proteintech, Rosemont, IL, USA). The protein bands were detected using an enhanced chemiluminescence (ECL) reagent (E1050, Lablead Biotechnology, Beijing, China), and protein expression levels were normalized to glyceraldehyde-3-phosphate dehydrogenase (GAPDH; 60004-1-Ig; Proteintech, Rosemont, IL, USA) as the loading control.

### 3.19. Real-Time Quantitative PCR Analysis

The pancreatic tissue, epididymal adipose tissue, and RAW264.7 cells were lysed with Trizol reagent and then centrifuged. Subsequently, chloroform was added to extract the RNA. The supernatant was gathered, and an equivalent volume of isopropanol was introduced to precipitate the RNA. The precipitate was rinsed with absolute ethanol and 75% ethanol. The RNA concentration was determined, and the reverse transcription reaction system was established to convert the RNA into cDNA. Reverse transcription was conducted using the Evo M-MLV cDNA Synthesis Kit (AG11307, AG, Wuhan, Hubei, China), followed by RT-PCR reactions with the PCR system kit (AG11707, AG, Wuhan, Hubei, China). The PCR reaction system comprises 1 µL cDNA, 0.8 µL primer, 3.2 µL water, and 5 µL SYBR Green. The quantitative real-time PCR amplification was performed under the following optimized conditions: initial denaturation at 95 °C for 30 s, followed by 40 cycles of denaturation at 95 °C for 5 s and combined annealing/extension at 60 °C for 30 s, with a final melt curve analysis stage ramping from 65 °C to 95 °C at an increment rate of 0.5 °C per second to verify amplification specificity. The relative mRNA expression levels were quantified using the comparative 2^−ΔΔCt^ method, where threshold cycle (Ct) values from the exponential amplification phase were used to first calculate ΔCt values (Ct_target gene, Ct_GAPDH reference gene) for each sample, followed by determination of ΔΔCt values (ΔCt_treated group, ΔCt_control group) to derive the relative fold change in gene expression (2^−ΔΔCt^), with all reactions performed in triplicate and data normalized to the internal control GAPDH before statistical analysis using GraphPad Prism software (veriosn 5.0). The primer sequences used in this study are listed in Table 1.

### 3.20. Statistical Analysis

Statistical analysis and graphical representation were performed using GraphPad Prism 5 software (Inc., San Diego, CA, USA). All continuous variables are presented as mean ± standard deviation (Mean ± SD). For between-group comparisons, normality was first assessed using the Shapiro–Wilk test. When data met both normality and homogeneity of variance (assessed by Bartlett’s test), two-group comparisons were analyzed by unpaired Student’s *t*-test, while Welch’s *t*-test was applied for data with unequal variances. Non-normally distributed data were analyzed using the nonparametric Mann–Whitney U test. For multiple group comparisons, one-way analysis of variance (ANOVA) was employed for normally distributed data with homogeneous variances, followed by Bonferroni post hoc tests when significant differences were detected (*p* < 0.05). The nonparametric Kruskal–Wallis test with Dunn’s post hoc correction was used when assumptions of normality or homogeneity of variance were violated. All tests were two-tailed, with statistical significance set at *p* < 0.05.

## 4. Discussion

In AP, pancreatic function impairment is accompanied by widespread dysfunction of immune cells. When the condition progresses to SAP, extensive necrosis occurs in pancreatic tissue, accompanied by systemic inflammatory responses, ultimately leading to multiple organ dysfunction [30,31]. Among these, acute kidney injury is a common complication in patients with SAP [32]. In this study, H&E staining found renal tubule necrosis, glomerular sac adhesion, acinar septum expansion, and acinar cell swelling and necrosis in obesity-related SAP mice, which further confirmed the occurrence of SAP. Simultaneously, the pathological damage to epididymal adipose tissue was most pronounced in the obesity-related SAP group. Additionally, the study results showed that obesity significantly increased the activity of serum amylase and lipase in SAP mice and markedly elevated the levels of multiple pro-inflammatory factors in the serum. Obesity also significantly increased the levels of various pro-inflammatory factors in pancreatic tissue, epididymal adipose tissue, and ATMs. These results collectively indicate that obesity significantly exacerbates systemic inflammatory responses and multi-organ damage during SAP. Notably, previous studies [19,33], combined with our experimental findings, have confirmed a significant increase in the infiltration of M1-type macrophages in obesity-associated epididymal adipose tissue, suggesting that ATMs may be a critical source of systemic inflammatory responses under disease conditions.

Oxidative stress is recognized as a pivotal factor in the early stages of AP [34]. Under normal physiological conditions, the pancreatic tissue maintains a delicate balance between oxidation and antioxidation [35]. However, when there is an abnormal increase in the level of ROS, it exacerbates the activation of inflammatory signals, thereby amplifying the inflammatory response and leading to pancreatic injury [36]. A previous study has reported that in rat models of AP induced by HFD, there is an elevation in MDA level within the pancreatic tissue, accompanied by a decrease in SOD level [37]. Consistent with the previous study, our data indicate that obese individuals exhibit significantly elevated levels of oxidative stress in serum, pancreatic tissue, epididymal adipose tissue, and ATMs during SAP. Thus, these results suggest that obesity may further exacerbate the systemic inflammatory response and tissue damage during SAP by intensifying oxidative stress reactions.

The core mechanism of ferroptosis lies in iron overload and lipid peroxidation [8]. Ferritin serves as the primary regulator of intracellular iron, ensuring physiological iron concentration balance by binding iron ions to prevent potential harm from iron overload [38]. Excessive free iron, catalyzed by the Fenton reaction, generates harmful ROS, which are the primary culprits in ferroptosis [39]. Meanwhile, the depletion of glutathione and inactivation of GPX4 lead to the accumulation of lipid peroxidation, whose metabolic products, such as MDA and 4-HNE, disrupt the structural integrity of cell membranes and the functional integrity of proteins [40]. Studies have confirmed a close association between AP and abnormal increases in iron levels and ROS levels in the pancreas [41]. Previous research has found that experimentally induced SAP in rats can result in the accumulation of iron, the enhancement of lipid peroxidation, and an upregulation of proteins associated with iron metabolism in the pancreas [42]. Notably, studies have shown that Lip-1 intervention can significantly inhibit the progression of SAP in rats [43]. In this study, our results found that obesity exacerbates ferroptosis in pancreatic tissue, epididymal adipose tissue, and ATMs during SAP by increasing levels of iron and lipid peroxidation. Lip-1 effectively alleviates lipid peroxidation and iron overload, inhibiting ferroptosis and subsequently reducing inflammation. Furthermore, this study employed immunofluorescence to detect the expression of F4/80 and 4-HNE in epididymal adipose tissue and ATMs. Immunofluorescence results demonstrated that ferroptosis can occur in macrophages within adipose tissue. Furthermore, in vitro experiments confirmed that ATMs undergo ferroptosis during obesity-related SAP, and the use of Lip-1 significantly alleviated inflammatory responses. These findings highlight the potential therapeutic value of the ferroptosis inhibitor Lip-1 in treating obesity-related SAP.

CD36, acting as a transporter for fatty acids and oxidized lipids, facilitates the transfer of various lipid types [44,45] and is highly expressed in hyperlipidemic pancreatitis [46]. Previous research has shown that CD36 promotes the transport of oxLDL into double-negative regulatory T cells, inducing ferroptosis in these cells, which subsequently exacerbates liver immune homeostasis disruption and the progression of metabolic dysfunction-associated steatotic liver disease [47]. Furthermore, other research has reported that CD36 promotes ferroptosis and inflammatory responses in CD4+ T cells following acute artery injury, while inhibiting CD36 can enhance CD4+ T cell function and improve the prognosis of patients with acute type A aortic dissection [48]. CD36 also plays a crucial regulatory role in oxidative stress and inflammatory responses within macrophages. In atherosclerosis, CD36 promotes the entry of long-chain fatty acids into the mitochondria of macrophages, impairing the function of Complex V (ATP synthase) in the mitochondrial electron transport chain. This damage leads to excessive generation of mitochondrial-derived ROS, which subsequently activates the NF-κB signaling pathway, triggering the release of a large number of inflammatory factors. Additionally, metabolic reprogramming in macrophages further induces the polarization of macrophages from the anti-inflammatory M2 phenotype to the pro-inflammatory M1 phenotype [49].

However, to date, there have been limited studies linking CD36 to obesity-related SAP. In our study, we observed a significant increase in CD36 protein levels in pancreatic tissue, epididymal adipose tissue, and ATMs in obesity-related SAP. Based on these findings, we hypothesize that CD36 may serve as a pivotal factor in exacerbating ferroptosis in obesity-related SAP. To substantiate this hypothesis, our study employed the CD36 inhibitor SSO for further validation. The results suggest that SSO effectively mitigates changes in ferroptosis and inflammatory markers in obesity-related SAP. Hence, our results suggest that CD36 induces inflammation by the ACSL4/GPX4 axis in pancreatic tissue, while the FTH1/GPX4 axis plays a similar regulatory role in epididymal adipose tissue and ATMs.

However, our study is not without its limitations. Notably, the constraints on sampling materials precluded the use of human tissue samples for verification. Additionally, technical limitations hindered our ability to culture macrophages from the adipose tissue of mice. In light of these factors, future studies should endeavor to address these shortcomings in order to validate our conclusions with a higher degree of certainty.

## 5. Conclusions

This study is the first to demonstrate that obesity exacerbates ferroptosis and inflammatory responses in pancreatic tissue, epididymal adipose tissue, and ATMs during SAP. The use of the ferroptosis inhibitor Lip-1 significantly alleviated these inflammatory responses. Furthermore, in obesity-related SAP, CD36 promotes ferroptosis in pancreatic tissue, epididymal adipose tissue, and ATMs, thereby inducing inflammatory responses. The use of the CD36 inhibitor SSO significantly mitigated these changes. This study elucidates the critical role of CD36 in obesity-associated SAP and suggests that inhibiting CD36 function may provide a novel potential strategy for the prevention and treatment of this disease.

## Figures and Tables

**Table 1:** Gene and Corresponding RT-qPCR Primer Sequences.

Gene	Forward Primer (5′→3′)	Reverse Primer (5′→3′)
IL-6	TAGTCCTTCCTACCCCAATTTCC	TTGGTCCTTAGCCACTCCTTC
IL-1β	GCAACTGTTCCTGAACTCAACT	ATCTTTTGGGGTCCGTCAACT
TNF-α	CCCTCACATCAGATCATCTTCT	GCTACGACGTGGGCTACAG
iNOS	GTTCTCAGCCCAACAATACAAGA	GTGGACGGGTCGATGTCAC
CD86	CTGGAAACGGAGACAGATACAC	TTAGGTGACTTTGGTCTCCGTT
CD206	CTCTGTTCAGCTATTGGACGC	CGGAATTTCTGGGATTCAGCT
GAPDH	AGGTCGGTGTGAACGGATTTG	TGTAGACCATGTATGAGGTCA

## Data Availability

Data used during the study are available from the corresponding author by reasonable request.

## Supplementary Materials

The following supporting information can be downloaded at: https://www.mdpi.com/article/10.3390/ijms26083482/s1.
