# Supplementary material for: CD36 Induces Inflammation by Promoting Ferroptosis in Pancreas, Epididymal Adipose Tissue, and Adipose Tissue Macrophages in Obesity-Related Severe Acute Pancreatitis
Source: Int J Mol Sci. 2025 Apr 8;26(8):3482. doi: 10.3390/ijms26083482 (PMC12027088; doi:10.3390/ijms26083482)
Supplement: Supplementary file 1 [file ijms-26-03482-s001.zip › ijms-3539415-supplementary.pdf]

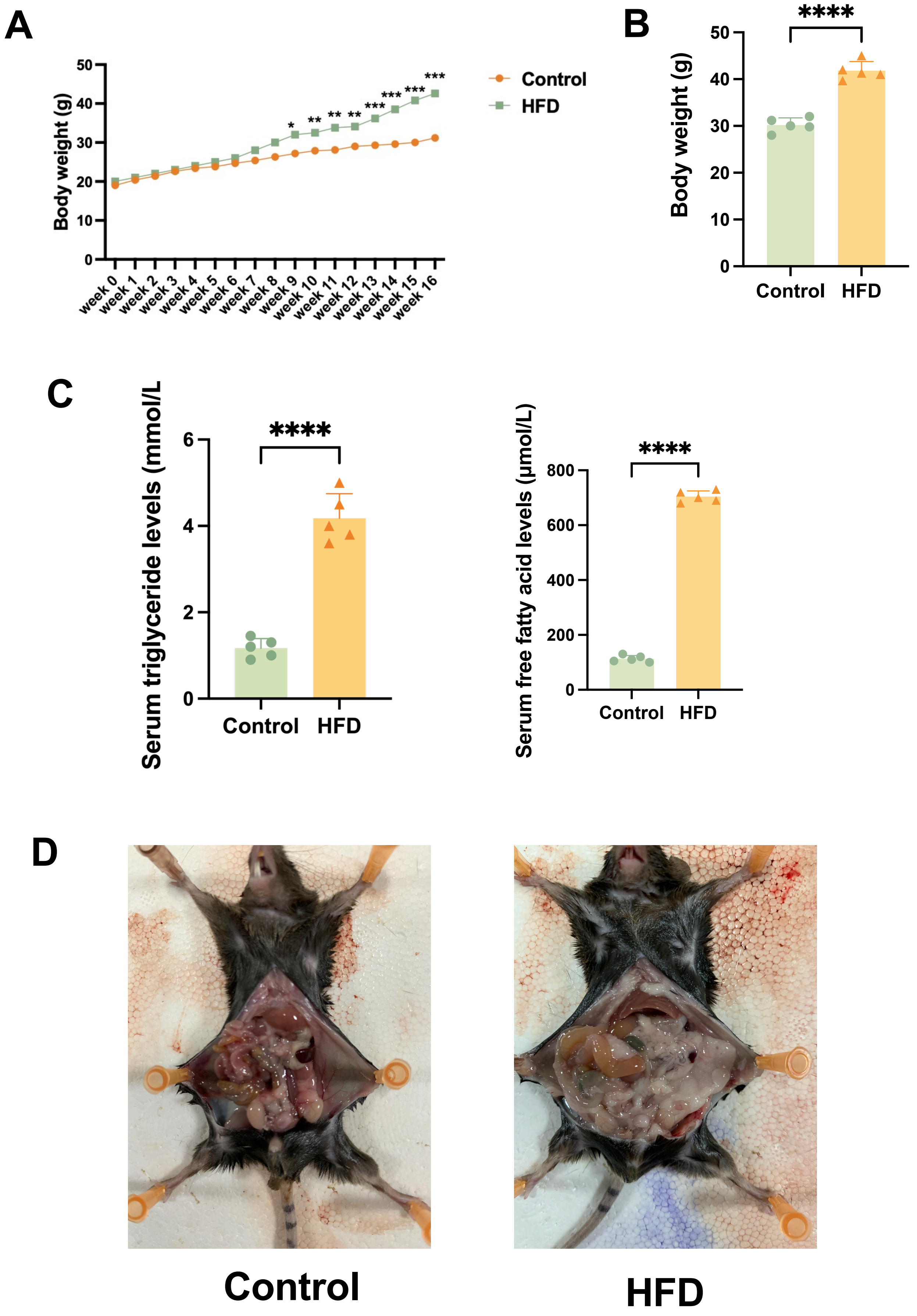

Supplementary Figure S1. Establishment of the obese mouse model. (A) Line graph of body weight changes in Control and HFD mice from the start of feeding with standard chow or high-fat diet (0-16 weeks). (B) After 16 weeks of feeding, a significant difference in average body weight was observed between the Control and HFD groups.(C) Serum triglyceride and free fatty acid levels in Control and HFD mice at 16 weeks. (D) The image shows that after 16 weeks of feeding, the epididymal adipose tissue volume in the HFD group mice is significantly larger than that in the Control group mice.
